# Building a framework for integrative longevity science: from rediscovery to innovation

**DOI:** 10.3389/fragi.2026.1742582

**Published:** 2026-05-19

**Authors:** Marta Gonzalez-Freire

**Affiliations:** Translational Research in Aging and Longevity Group (TRIAL Group), Health Research Institute of the Balearic Islands (IdISBa), Palma de Mallorca, Spain

**Keywords:** aging biomarkers, biological age, cardiorespiratory fitness, digital health, geroscience, healthspan, longevity, wearable Technology

## Abstract

The longevity research field is experiencing unprecedented momentum, driven by advances in molecular biomarkers, digital health technologies, and artificial intelligence. However, established physiological measures—VO_2_max, heart rate variability, body composition, muscle strength, among others—with decades of validation are increasingly presented as novel “longevity biomarkers” without acknowledging their foundational research. This perspective proposes integration as the constructive path forward, creating multidimensional aging models that combine functional measures, molecular biomarkers, mechanistic studies, and longitudinal outcomes. We present a practical framework across five domains—endpoint definition, biomarker validation, integration strategy, study design, and communication—to guide the field from rediscovery to meaningful innovation. Longevity science’s future depends on methodological rigor, transparent communication, and genuine integration of established knowledge with novel insights, ensuring that enthusiasm for new technologies translates into demonstrable improvements in healthspan and potentially lifespan.

## Introduction

The longevity research landscape is at a pivotal moment. Novel molecular biomarkers, digital health technologies, artificial intelligence platforms, and candidate interventions are converging with unprecedented momentum, attracting interest from scientists, clinicians, investors, and the public. This enthusiasm presents both an opportunity and a challenge: how to advance longevity science in ways that are both ambitious and methodologically rigorous, ensuring credibility grows in step with discovery.

However, a concerning pattern has emerged. Established health metrics with decades of validation—such as maximal oxygen uptake (VO_2_ max), resting heart rate (RHR), heart rate variability (HRV), body composition assessment, and muscle strength—are increasingly presented as newly discovered “longevity biomarkers” in consumer applications, startup pitches, and even peer-reviewed publications. Their predictive value for morbidity and mortality is well established (Blair et al., 1989; Kodama et al., 2009; Rantanen et al., 2000; Cooper et al., 2010; Tsuji et al., 1994), yet this foundation is sometimes obscured rather than built upon. The challenge is not to rebrand what is already well known, but to integrate physiological measures with molecular biomarkers and mechanistic insights to advance the field meaningfully.

## The rebranding phenomenon: specific examples

Recent examples illustrate this pattern across multiple domains.

### Consumer wearables and “aging scores”

Several direct-to-consumer longevity companies now market wearable-derived metrics—including HRV, resting heart rate, sleep stages, and activity patterns—as “biological age” assessments or “aging velocity” measures. While these physiological parameters do correlate with health outcomes, their repackaging as novel aging biomarkers often lacks transparent validation against established aging clocks or longitudinal health outcomes. A 2022 review of omics-based biological age measures found substantial heterogeneity in what commercial algorithms measure, with many showing poor agreement with validated epigenetic clocks and clinical outcomes (Rutledge et al., 2022). Similarly, body composition measures—assessed via DXA or bioimpedance—have long predicted age-related health outcomes, yet are now marketed as novel “metabolic age” indicators by consumer devices. The value proposition rests on frequency of measurement and AI integration, not on discovering previously unknown health indicators.

### Continuous glucose monitoring beyond diabetes

CGMs, long central to diabetes management, are now marketed to metabolically healthy individuals as longevity optimization tools. Companies position glycemic variability metrics as indicators of “metabolic age” or aging rate. Yet the evidence linking glucose variability in normoglycemic individuals to aging biology remains preliminary (Hall et al., 2018), and guidelines do not support CGM use in individuals without diabetes or prediabetes. Notably, the PREDICT studies demonstrated substantial inter-individual variability in postprandial glycemic responses to identical foods, even among healthy individuals (Berry et al., 2020), underscoring that population-derived “optimal ranges” may not apply to individual optimization. This represents a substantial expansion beyond the evidence base.

### VO_2_ max and cardiorespiratory fitness

Perhaps the most striking example: VO_2_ max has been rigorously documented as one of the strongest predictors of all-cause mortality since the 1980s (Blair et al., 1989; Kodama et al., 2009). Some recent longevity-focused publications and platforms present cardiorespiratory fitness metrics as if their predictive value were a recent discovery, overlooking decades of exercise physiology and epidemiological research that established these relationships.

These examples share a common thread: established physiological measures are being recontextualized—often with sophisticated digital interfaces and AI-driven dashboards—but without always acknowledging their historical foundation or clearly delineating what is genuinely novel versus what is repackaged. This approach risks undermining scientific credibility and creating confusion among clinicians, policymakers, and consumers about where genuine innovation lies.

## From rediscovery to integration: a constructive path

**FIGURE 1:**
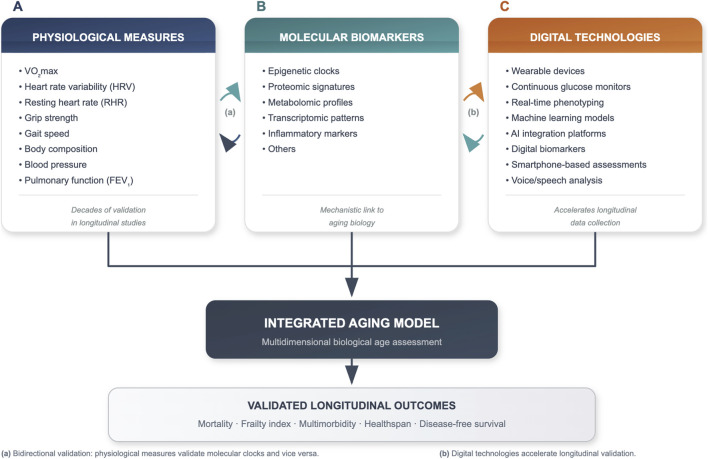
Integrative framework for longevity science: from established foundations to multidimensional innovation. Integrative framework illustrating three complementary domains of aging assessment. Panel **(A)** Physiological measures with decades of validation, including VO_2_max, heart rate variability (HRV), resting heart rate (RHR), grip strength, gait speed, body composition, blood pressure, and pulmonary function (FEV_1_). Panel **(B)** Molecular biomarkers such as epigenetic clocks, proteomic signatures, metabolomic profiles, transcriptomic patterns, inflammatory markers, and others. Panel **(C)** Digital technologies including wearable devices, continuous glucose monitors, real-time phenotyping, machine learning models, AI platforms, digital biomarkers, smartphone-based assessments, and voice/speech analysis. Bidirectional arrows indicate reciprocal validation between domains: physiological measures validate molecular clocks and vice versa **(a)**, while digital technologies accelerate longitudinal data collection **(b)**. All domains converge toward an integrated aging model validated against longitudinal outcomes (mortality, frailty, multimorbidity, healthspan). This framework emphasizes building on established knowledge while incorporating novel technologies, ensuring scientific credibility through transparent validation and mechanistic linkage to aging biology.

The solution is not to diminish enthusiasm for digital health and AI-enabled monitoring, nor to dismiss the value of more frequent, granular phenotyping. Rather, the opportunity lies in integration: combining well-validated functional measures with validated molecular biomarkers of aging, mechanistic studies, and longitudinal outcome data to create truly multidimensional models (Figure 1).

The promise of wearables and AI is real—but it rests on population-scale application, rigorous validation, and transparent linkage to aging biology, not on rediscovering metrics that exercise physiologists established decades ago. Similarly, molecular aging biomarkers—epigenetic clocks, proteomic signatures, metabolomic profiles—offer genuine mechanistic insight (Horvath and Raj, 2018), but they require continued standardization, validation across diverse populations, and clinical anchoring to outcomes (Ferrucci et al., 2020).

Critically, AI and machine learning represent not merely drivers of the rebranding problem, but essential methodological tools for solving the integration challenge. The relationships between molecular aging clocks and physiological function are non-linear and context-dependent—patterns that traditional statistical approaches struggle to capture. Machine learning algorithms can identify complex interactions across data types, detect early signatures of accelerated aging, and generate personalized risk trajectories (Earls et al., 2019; Galkin et al., 2020). However, the field must prioritize interpretable models over black-box predictions: clinicians and patients need to understand why an algorithm flags concern, not merely that it does. Open-source validation efforts and transparent reporting of algorithm performance across diverse populations will be essential to building trust and ensuring equitable benefit.

## Interpreting multi-domain discordance

A truly integrative framework must address a common clinical scenario: discordance between different biomarker domains. An individual may present with a “young” epigenetic age but poor cardiorespiratory fitness, or conversely, excellent VO_2_ max despite accelerated molecular aging markers. Such discordance is not a failure of measurement but rather reflects the biological reality of “mosaic aging”—the recognition that different organ systems and biological processes age at different rates within the same individual (Oh et al., 2023; Ahadi et al., 2020).

Rather than viewing discordance as problematic, clinicians and researchers should interpret it as clinically actionable information. When physiological function lags behind molecular age, targeted exercise and lifestyle interventions may be prioritized. When molecular markers suggest accelerated aging despite maintained function, closer monitoring and investigation of subclinical processes may be warranted. Indeed, discordance itself may serve as a novel biomarker—potentially indicating specific system vulnerabilities or compensatory mechanisms that warrant investigation (Li et al., 2020). The Dunedin Pace of Aging studies have demonstrated that biological aging rates vary substantially across individuals and can be modified by intervention, supporting the utility of repeated multi-domain assessment (Belsky et al., 2022).

## Clarifying healthspan and lifespan

A related conceptual challenge is the frequent conflation of healthspan and lifespan. Healthspan—years lived in good health—clearly benefits from exercise, nutrition, sleep, and metabolic optimization; decades of research support morbidity compression and functional preservation (Fries, 1980). Whether such behavioral interventions extend maximum human lifespan remains uncertain. Epigenetic age acceleration reversal in interventional studies is promising (Fahy et al., 2019) but does not yet constitute evidence for lifespan extension.

Presenting improvements in VO_2_ max, HRV, or metabolic parameters as definitive evidence that “aging has been slowed” risks conflating functional improvements with deceleration of biological aging processes. Precision in endpoints and terminology strengthens both science and public trust.

## A framework for moving forward

To support the transition from rediscovery to meaningful integration, we propose a practical framework organized around five key domains (Table 1).

**TABLE 1:** Framework for integrative longevity science: moving from rediscovery to innovation.

Domain	Specific recommendations
Endpoint definition	• Distinguish healthspan from lifespan explicitly in study design and reporting • Specify whether interventions target functional capacity, disease risk, or biological aging mechanisms • Use standardized outcome definitions aligned with geroscience frameworks (e.g., NIH geroscience assessments)
Biomarker validation	• For molecular measures: Require reproducibility, validation in diverse cohorts, and clinical outcome anchoring (mortality, frailty, multimorbidity) • For wearable-derived metrics: Adopt reporting standards (device specifications, sampling frequency, artifact handling algorithms) and assess cross-device consistency • Report predictive performance transparently, including confidence intervals and overfitting assessments
Integration strategy	• Combine physiological capacity measures (VO_2_ max, strength, HRV) with validated biological aging measures to create multidimensional models, rather than positioning any single legacy metric as a standalone “aging score” • Build on existing knowledge: cite foundational work establishing predictive value of functional measures before claiming novelty • Develop composite scores only after demonstrating added value over individual components
Study design	• Prioritize longitudinal cohorts with sufficient follow-up to capture aging trajectories and outcomes • Include mechanistic endpoints linking interventions to aging biology (mitochondrial function, cellular senescence, inflammation) • Design trials aligned with geroscience frameworks testing whether interventions alter pace of biological aging, not just disease risk
Communication	• State limitations, confidence intervals, and plausible effect sizes when translating findings to consumers, clinicians, or policymakers • Distinguish between established associations (e.g., VO_2_ max and mortality) and emerging hypotheses (e.g., epigenetic age reversal and lifespan) • Acknowledge historical context and foundational research when presenting biomarkers or technologies

## Discussion

Longevity science has an opportunity to deliver substantial public benefit: not only more years of life, but also more years in good health. Realizing that promise depends on building credibility through methodological rigor, transparent communication, and genuine integration of established knowledge with novel insights.

Wearables, AI platforms, and continuous monitoring technologies can accelerate progress—but only if their outputs are validated transparently and connected rigorously to aging biology. Exercise, nutrition, and lifestyle factors remain the most powerful population-level levers for healthy aging (Booth et al., 2012; Estruch et al., 2013), not because they are “discoveries,” but because they are proven foundations. The future of longevity science lies in integrating these pillars with validated molecular biomarkers, mechanistic studies, and targeted interventions that demonstrably modify aging trajectories.

By adopting shared standards, maintaining conceptual precision, and communicating uncertainty responsibly, the longevity field can match its considerable ambition with durable credibility. The question is not whether we can extend healthspan and lifespan—it is how we build the evidence base to do so with scientific integrity and public trust.

An often-overlooked dimension of this challenge is accessibility. Many “rebranded” longevity metrics are hidden behind expensive paywalls or proprietary wearable algorithms, whereas the foundational physiological tests they derive from—grip strength, walking speed, simple cardiorespiratory assessments—are low-cost and globally accessible. As the longevity field matures, there is an ethical imperative to ensure that integrative aging assessment does not become a luxury consumer product available only to affluent early adopters. Open-source validation efforts, transparent algorithm reporting, and investment in low-cost assessment tools will be essential to democratizing the benefits of longevity science across socioeconomic strata and global populations (Braveman and Gottlieb, 2014).

Finally, preventing future “rediscoveries” requires rethinking how we train longevity scientists. The current siloed structure of academic training—where molecular biologists rarely engage with exercise physiology, and data scientists may lack clinical context—contributes to the reinvention of established knowledge. The field would benefit from interdisciplinary training programs that produce researchers equally comfortable with epigenetic clocks and cardiopulmonary exercise testing, with machine learning pipelines and longitudinal cohort design. Such “integrative longevity scientists” would be better positioned to build on existing foundations rather than inadvertently rebrand them.

## Data Availability

The original contributions presented in the study are included in the article/supplementary material, further inquiries can be directed to the corresponding author.
